# Supplementary material for: Carry-Over of Zearalenone and Its Metabolites to Intestinal Tissues and the Expression of CYP1A1 and GSTπ1 in the Colon of Gilts before Puberty
Source: Toxins (Basel). 2022 May 18;14(5):354. doi: 10.3390/toxins14050354 (PMC9145504; doi:10.3390/toxins14050354)
Supplement: Supplementary file 1 [file toxins-14-00354-s001.zip › toxins-1713282-supplementary.pdf]

toxins

MDPI

Supplementary Materials: Carry-Over of Zearalenone and Its Metabolites to Intestinal Tissues and the Expression of CYP1A1 and GSTπ1 in the Colon of Gilts before Puberty

Magdalena Mróz, Magdalena Gajęcka, Paweł Brzuzan, Sylwia Lisieska-Żołnierczyk, Dawid Leski, Łukasz Zielonka and Maciej T. Gajęcki

Task 4. Validation of chromatographic determination methods for zearalenone, α-zearalenol and DON in pig feed

Validation of the zearalenone (ZEN) determination method in wheat

The main points of QC-Quality Control:

LQC–low quality control–10 µg/kg

IQC–intermediate quality control–40 µg/kg

MQC–medium quality control–100 µg/kg

HQC–high quality kontrol–160 µg/kg

Method range: 5–200 µg/kg

1. Determination of precision and accuracy

LQC – 10 µg/kg

Parameters	ZEN concentration µg/kg		
Average concentration on the day	9.68	9.69	10.08
SD	0.32	0.29	0.38
CV	3.27	3.04	3.82
Bias %	-3.16	-3.10	0.80
Number of samples	5	5	5
Mean total concentration	9.82		
Total SD	0.36		
CV % total	3.71		
Bias% total	-1.82		
Number of samples	15		

Variant	Sum of squares	Number of degrees of freedom	Variance	F	p
During the day	0.51	2	0.25	2.30	0.14
Between days	1.34	12	0.11		
Total	1.85	14	0.36		

IQC – 40 µg/kg

Parameters	ZEN concentration µg/kg		
Average concentration on the day	39.65	39.92	39.78

**SD**	2.30	0.80	1.37
**CV**	0.75	2.01	3.44
**Bias %**	-0.86	-0.21	0.55
**Number of samples**	5	5	5
**Mean total concentration**	39.78		
**Total SD**	0.87		
**CV % total**	2.19		
**Bias% total**	-0.54		
**Number of samples**	15		

Variant	Sum of squares	Number of degrees of freedom	Variance	F	p
**During the day**	0.17	2	0.08	0.09	0.90
**Between days**	10.43	12	0.86		
**Total**	10.60	14	0.95		

MQC – 100 µg/kg

Parameters	ZEN concentration µg/kg		
**Average concentration on the day**	91.52	95.83	101.23
**SD**	3.92	4.49	3.20
**CV**	4.28	4.69	3.17
**Bias %**	-8.48	-4.17	1.23
**Number of samples**	5	5	5
**Mean total concentration**	96.19		
**Total SD**	5.47		
**CV % total**	5.69		
**Bias% total**	-3.81		
**Number of samples**	15		

Variant	Sum of squares	Number of degrees of freedom	Variance	F	p
**During the day**	236.30	2	118.15	7.73	0.00
**Between days**	183.30	12	15.27		
**Total**	419.60	14	133.42		

HQC – 160 µg/kg

Parameters	ZEN concentration µg/kg

**Average concentration on the day**	158.47	163.23	157.52
**SD**	2.79	3.76	4.88
**CV**	1.76	2.30	3.10
**Bias %**	-0.95	2.02	-1.55
**Number of samples**	5	5	5
**Mean total concentration**	159.74		
**Total SD**	4.44		
**CV % total**	2.78		
**Bias% total**	-0.16		
**Number of samples**	15		

Variant	Sum of squares	Number of degrees of freedom	Variance	F	p
**During the day**	93.62	2	46.81	3.06	0.08
**Between days**	183.07	12	15.25		
**Total**	276.70	14	62.06		

2. Determination of linearity – study of linearity and bias

Correlation coefficient – 0.99

**Regression table**					
**Parameter**	**Value**	**Standard error**	**Statistics t**	**p**	
**Free word**	-0.61	0.82	-0.73	0.46	
**Inclination**	-0.00	0.00	-0.76	0.44	
**Table ANOWA**					
**Source**	**Sum of squares**	**Degrees of freedom**	**Medium square**	**F**	**p**
**Regression**	8.48	1	8.48	0.58	0.44
**Error**	845.03	58	14.56		
**Together**	853.52	59			
**Materiality of a systemic error**					
**Part**	**Reference value**	**Average of measurements**	**Systematic error**	**p**	
**1**	10	9.82	-0.18	0.09	
**2**	40	39.78	-0.21	0.44	
**3**	100	96.19	-3.80	0.01	
**4**	160	159.74	-0.26	0.84	
**Average**			-1.11		

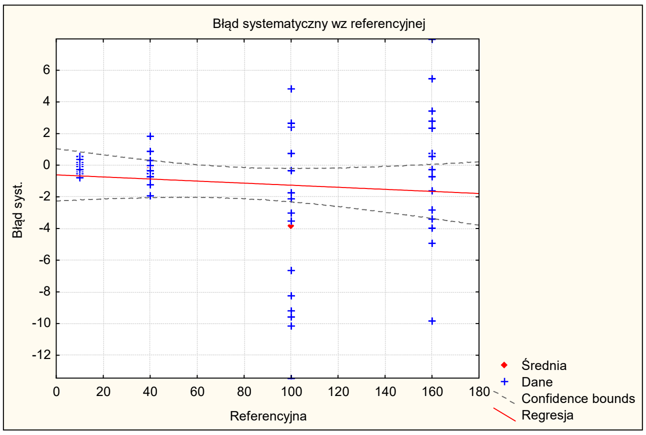

3. Determination of recovery

Nominal concentration ZEN µg/kg	Total ZEN recovery %	Average total recovery %	CV of total recovery
**LQC**	96.84	98.18	3.71
	96.90		
	100.80		
**IQC**	99.13	99.46	2.19
	99.79		
	99.45		
**MQC**	91.52	96.19	5.69
	95.83		
	101.23		
**HQC**	99.04	99.84	2.78
	102.02		
	98.45		
	Average	98.42	3.59

4. Determination LOD - limit of detection

The limit of detection was determined on the basis of 7 repetitions of the blank sample (ZEN-free serum). Then the average value was calculated and the value of 3 standard deviations was calculated. The limit of detection was the sum of the mean values and 3 standard deviations. The limit of detection was 2 µg/kg.

5. Determination LOQ - the lowest limit of the quantitation

The limit of quantification was determined on the basis of 7 repetitions of the blank sample (ZEN-free serum). Then the average value was calculated and the value of 10 standard deviations was calculated. The limit of quantification is the sum of the mean values and 10 standard deviations. The limit of quantification was 5 μg/kg.

Calibration of the method for the determination of α-zearalenol (α-ZEL) in wheat

The main points of QC-Quality Control:

LQC–low quality control–10 μg/kg

IQC–intermediate quality control–40 μg/kg

MQC–medium quality control–100 μg/kg

HQC–high quality kontrol–160 μg/kg

Method range: 5–200 μg/kg

1. Determination of precision and accuracy

LQC – 10 μg/kg

Parameters	Concentration α-ZEL μg/kg		
**Average concentration on the day**	10.49	9.88	10.22
**SD**	0.21	0.33	0.55
**CV**	2.09	3.33	5.36
**Bias %**	4.94	-1.18	2.18
**Number of samples**	5	5	5
**Mean total concentration**	10.20		
**Total SD**	0.44		
**CV % total**	4.36		
**Bias% total**	1.98		
**Number of samples**	15		

Variant	Sum of squares	Number of degrees of freedom	Variance	F	p
During the day	0.93	2	0.46	3.08	0.08
Between days	1.82	12	0.15		
Total		14			

IQC – 40 μg/kg

Parameters	Concentration α-ZEL μg/kg		
Average concentration on the day	43.60	42.25	41.82
SD	0.99	0.92	0.97
CV	2.26	2.17	2.31
Bias %	9.00	5.63	4.55
Number of samples	5	5	5
Mean total concentration	42.56		
Total SD	1.18		
CV % total	2.78		
Bias% total	6.39		
Number of samples	15		

Variant	Sum of squares	Number of degrees of freedom	Variance	F	p
During the day	8.64	2	4.32	4.71	0.03
Between days	11.00	12	0.91		
Total	19.64	14	5.23		

MQC – 100 μg/kg

Parameters	Concentration α-ZEL μg/kg		
Average concentration on the day	100.26	95.62	98.15
SD	2.78	4.94	4.04
CV	2.77	5.17	4.11
Bias %	0.26	-4.38	-1.85
Number of samples	5	5	5
Mean total concentration	98.01		
Total SD	4.21		
CV % total	4.29		
Bias% total	-1.99		
Number of samples	15		

Variant	Sum of squares	Number of degrees of freedom	Variance	F	p
**During the day**	53.92	2	26.96	1.66	0.22
**Between days**	193.84	12	16.15		
**Total**	247.76	14	43.11		

HQC – 160 µg/kg

Parameters	Concentration α-ZEL µg/kg		
**Average concentration on the day**	162.59	158.87	156.01
**SD**	3.40	4.05	2.47
**CV**	2.09	2.55	1.58
**Bias %**	1.62	-0.71	-2.49
**Number of samples**	5	5	5
**Mean total concentration**	159.16		
**Total SD**	4.18		
**CV % total**	2.63		
**Bias% total**	-0.53		
**Number of samples**	15		

Variant	Sum of squares	Number of degrees of freedom	Variance	F	p
**During the day**	108.93	2	54.46	4.79	0.02
**Between days**	136.32	12	11.36		
**Total**	245.26	14	65.82		

2. Determination of linearity – study of linearity and bias

Correlation coefficient – 0.99

Regression table					
**Parameter**	**Value**	**Standard error**	**Statistics t**	**p**	
**Free word**	1.28	0.71	0.80	0.07	
**Inclination**	-0.01	0.00	-2.28	0.02	
**Table ANOWA**					
**Source**	**Sum of squares**	**Degrees of freedom**	**Medium square**	**F**	**p**
**Regression**	56.45	1	56.45	5.21	0.02
**Error**	627.73	58	10.82		
**Together**	684.19	59	67.28		
**Materiality of a systemic error**					

Part	Reference value	Average of measurements	Systematic error	p	
**1**	10	10.20	-0.19	0.05	
**2**	40	42.56	2.55	0.00	
**3**	100	98.01	-1.99	0.07	
**4**	160	159.16	-0.84	0.39	
**Average**			-0.01		

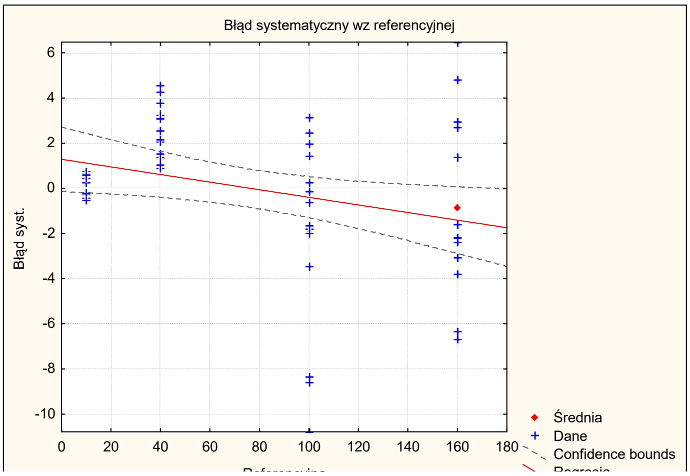

3. Determination of recovery

Nominal concentration α-ZEL µg/kg	Total recovery α-ZEL %	Average total recovery %	CV of total recovery
**LQC**	104.94	101.98	4.36
	98.82		
	102.18		
**IQC**	109.00	106.39	2.74
	105.63		
	104.55		
**MQC**	100.26	98.01	4.29

	95.62		
	98.15		
**HQC**	101.62	99.47	2.63
	99.29		
	97.51		
	Average	101.46	3.50

4. Determination LOD - limit of detection

The limit of detection was determined on the basis of 7 repetitions of the blank sample (wheat free of α-ZEL). Then the average value was calculated and the value of 3 standard deviations was calculated. The limit of detection was the sum of the mean values and 3 standard deviations. The limit of detection was 3 µg/kg.

5. Determination LOQ - the lowest limit of the quantitation

The limit of quantification was determined on the basis of 7 repetitions of the blank sample (wheat free of α-ZEL). Then the average value was calculated and the value of 10 standard deviations was calculated. The limit of quantification is the sum of the mean values and 10 standard deviations. The limit of quantification was 5 µg/kg.

Validation of the method for the determination of deoxynivalenol (DON) in wheat

The main points of QC-Quality Control:
LQC–low quality control–100 µg/kg
IQC–intermediate quality control–500 µg/kg
MQC–medium quality control–2500 µg/kg
HQC–high quality kontrol–4000 µg/kg
Method range: 25–5000 µg/kg

1. Determination of precision and accuracy

LQC – 100 µg/kg

Parameters	DON concentration µg/kg		
**Average concentration on the day**	107.00	103.56	106.65
**SD**	3.33	2.64	4.35
**CV**	3.12	2.55	4.08
**Bias %**	7.00	3.56	6.65
**Number of samples**	5	5	5
**Mean total concentration**	105.74		
**Total SD**	3.63		
**CV % total**	3.43		
**Bias% total**	5.74		
**Number of samples**	15		

Variant	Sum of squares	Number of degrees of freedom	Variance	F	p
**During the day**	35.95	2	17.98	1.45	0.27
**Between days**	148.30	12	12.36		
**Total**	184.25	14	30.34		

IQC – 500 µg/kg

Parameters	Concentration 500 µg/kg		
**Average concentration on the day**	508.78	502.83	501.67
**SD**	7.73	4.96	8.36
**CV**	1.52	0.99	1.67
**Bias %**	1.75	0.56	0.33
**Number of samples**	5	5	5
**Mean total concentration**	504.42		
**Total SD**	7.38		
**CV % total**	1.46		
**Bias% total**	0.88		
**Number of samples**	15		

Variant	Sum of squares	Number of degrees of freedom	Variance	F	p
**During the day**	145.39	2	72.70	1.41	0.28
**Between days**	617.00	12	51.42		
**Total**		14			

MQC – 2500 µg/kg

Parameters	Concentration 2500 µg/kg		
Average concentration on the day	2396.55	2423.85	2462.87
SD	55.22	37.57	25.32
CV	2.30	1.55	1.03
Bias %	-4.14	-3.05	-1.48
Number of samples	5	5	5
Mean total concentration	2427.76		
Total SD	47.45		
CV % total	1.95		
Bias% total	-2.89		
Number of samples	15		

Variant	Sum of squares	Number of degrees of freedom	Variance	F	p
During the day	11107.75	2	5553.87	3.26	0.07
Between days	20410.10	12	1700.84		
Total	31517.85	14	175637.87		

HQC – 4000 µg/kg

Parameters	DON concentration µg/kg		
Average concentration on the day	3063.04	3201.48	3190.96
SD	159.03	217.17	179.19
CV	5.19	6.78	5.61
Bias %	-23.42	-19.96	-20.23
Number of samples	5	5	5
Mean total concentration	3151.83		
Total SD	184.71		
CV % total	5.86		
Bias% total	-21.20		
Number of samples	15		

Variant	Sum of squares	Number of degrees of freedom	Variance	F	P
During the day	59399.15	2	29699.57	0.85	0.45
Between days	418271.20	12	34855.94		
Total	477670.35	14	64555.51		

2nd Determination of linearity – study of linearity and bias

Correlation coefficient – 0.999

Regression table					
**Parameter**	**Value**	**Standard error**	**Statistics t**	**p**	
**Free word**	122.65	40.30	3.04	0.00	
**Inclination**	-0.19	0.01	-11.61	0.00	
**Table ANOWA**					
**Source**	**Sum of squares**	**Degrees of freedom**	**Medium square**	**F**	**p**
**Regression**	5785472.84	1	5785472.84	134.89	0.00
**Error**	2487601.97	58	42889.68		
**Together**	8273074.81	59			
**Materiality of a systemic error**					
**Part**	**Reference value**	**Average of measurements**	**Systematic error**	**p**	
**1**	100	105.74	5.73	0.00	
**2**	500	504.42	4.42	0.03	
**3**	2500	2427.75	-72.24	0.00	
**4**	4000	3151.83	-848.17	0.00	
**Average**			-227.56		

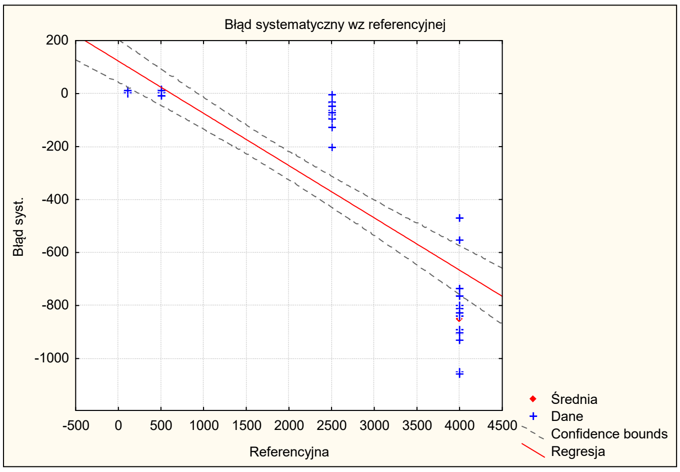

3. Determination of recovery

Nominal concentration of DON µg/kg	Total recovery of DON %	Average total recovery %	CV of total recovery
**LQC**	107.00	105.74	3.43
	103.56		
	106.65		
**IQC**	101.75	100.88	1.46
	100.56		
	100.33		
**MQC**	95.86	97.11	1.95
	96.95		
	98.51		
**HQC**	76.58	78.80	5.86
	80.04		
	79.77		
	Average	95.63	2.69

4. Determination LOD - limit of detection

The limit of detection was determined on the basis of 7 repetitions of a blank sample (DON-free wheat). Then the average value was calculated and the value of 3 standard deviations was calculated. The limit of detection was the sum of the mean values and 3 standard deviations. The limit of detection was 14 µg/kg.

5. Determination LOQ - the lowest limit of the quantitation

The limit of quantification was determined on the basis of 7 repetitions of the blank sample (DON-free wheat). Then the average value was calculated and the value of 10 standard deviations was calculated. The limit of quantification is the sum of the mean values and 10 standard deviations. The limit of quantification was 20 µg/kg.

Summary

The validation showed that in all tested control points for ZEN, α-ZEL and DON (LQC, IQC, MQC, HQC) within the batch (day of performing the determinations) and between batches (between days of determinations) the acceptance criteria for precision (BIAS% - systematic error of the method – did not exceed ±15%), accuracy (the coefficient of variation CV did not exceed ± 15%), the linearity of the method was maintained (correlation curve for each of

the analytes above r = 0.99), the average total % recovery was above 90%. For individual mycotoxins, the limit of detection (LOD) and the limit of quantification (LOQ) were determined.
